# The SIRT1/TP53 axis is activated upon B-cell receptor triggering via *miR-132* up-regulation in chronic lymphocytic leukemia cells

**DOI:** 10.18632/oncotarget.3905

**Published:** 2015-05-11

**Authors:** Michele Dal Bo, Tiziana D'Agaro, Stefania Gobessi, Antonella Zucchetto, Sara Dereani, Davide Rossi, Francesco Zaja, Gabriele Pozzato, Francesco Di Raimondo, Gianluca Gaidano, Luca Laurenti, Giovanni Del Poeta, Dimitar G. Efremov, Valter Gattei, Riccardo Bomben

**Affiliations:** ^1^ Clinical and Experimental Onco-Hematology Unit, Centro di Riferimento Oncologico, I.R.C.C.S., Aviano (PN), Italy; ^2^ Molecular Hematology, International Centre for Genetic Engineering and Biotechnology, Rome, Italy; ^3^ Division of Hematology, Department of Clinical and Experimental Medicine, Amedeo Avogadro University of Eastern Piedmont, Novara, Italy; ^4^ Clinica Ematologica, Centro Trapianti e Terapie Cellulari "Carlo Melzi" DISM, Azienda Ospedaliera Universitaria S. Maria Misericordia, Udine, Italy; ^5^ Department of Internal Medicine and Hematology, Maggiore General Hospital, University of Trieste, Trieste, Italy; ^6^ Division of Hematology, Ferrarotto Hospital, Catania, Italy; ^7^ Department of Hematology, Catholic University Hospital A. Gemelli, Rome, Italy; ^8^ Division of Hematology, S.Eugenio Hospital and University of Tor Vergata, Rome, Italy

**Keywords:** CLL, BCR, miR-132

## Abstract

The B-cell receptor (BCR) plays an important role in the pathogenesis and progression of chronic lymphocytic leukemia (CLL). By global microRNA profiling of CLL cells stimulated or not stimulated by anti-IgM, significant up-regulation of microRNAs from the *miR-132~212* cluster was observed both in *IGHV* gene unmutated (UM) and mutated (M) CLL cells. Parallel gene expression profiling identified *SIRT1*, a deacetylase targeting several proteins including TP53, among the top-ranked *miR-132* target genes down-regulated upon anti-IgM exposure. The direct regulation of SIRT1 expression by *miR-132* was demonstrated using luciferase assays. The reduction of *SIRT1* mRNA and protein (*P = 0.001*) upon anti-IgM stimulation was associated with an increase in TP53 acetylation (*P = 0.007*), and the parallel up-regulation of the TP53 target gene *CDKN1A*. Consistently, *miR-132* transfections of CLL-like cells resulted in down-regulation of SIRT1 and an induction of a TP53-dependent apoptosis. Finally, in a series of 134 CLL samples, *miR-132*, when expressed above the median value, associated with prolonged time-to-first-treatment in patients with M CLL (HR = 0.41; *P = 0.02*). Collectively, the *miR-132*/SIRT1/TP53 axis was identified as a novel pathway triggered by BCR engagement that further increases the complexity of the interactions between tumor microenvironments and CLL cells.

## INTRODUCTION

Chronic lymphocytic leukemia (CLL) is a neoplastic disease characterized by highly variable clinical courses, ranging from rapid progression with fatal outcome to a relatively indolent behaviour with normal life expectancy [1]. In this regard, a more aggressive clinical course has been associated with specific features of the B-cell receptor (BCR) expressed by CLL cells, particularly the so-called unmutated (UM) configuration, i.e. less than 2% point mutations, of the genes coding for the immunoglobulin heavy-chain variable (*IGHV*) region of the BCR [1].

The role of the BCR in the pathogenesis and progression of CLL is also suggested by the following observations: i) about 20% of CLL use biased groups of *IGHV* genes expressing similar/identical HCDR3s and identical light chains (stereotyped BCRs), indicating that the initial clonal expansions can be antigen-driven [2–5]; ii) a strong correlation is observed between the clinical course of CLL and expression of certain stereotyped BCRs [2, 4, 5]; iii) freshly isolated CLL cells show increased expression of BCR target genes and reduced expression of surface IgM, suggesting continuous antigen stimulation *in vivo* [6–8]. Moreover, the promising results of clinical trials with agents targeting the BCR signaling pathway, such as inhibitors of SYK, BTK, and PI3Kδ, again indicate that chronic BCR signaling is required for CLL cell growth and survival [9–12]. It is worth noting, however, that CLL BCRs also display features of auto-reactivity, their engagement potentially triggering signaling cascades leading to anergy and/or apoptosis, resulting in cell death rather than increased survival [13–20]. What outcome will predominate is determinate by several factors, such as BCR signal intensity, BCR signal duration, and availability of co-stimulatory signals [21–23].

MicroRNAs represent a class of small non-coding RNAs that act as master regulators of protein expression by inhibiting the translation or inducing the degradation of target mRNAs with partially complementary sites in the 3′-untranslated regions (3′-UTR) [24]. In cell patho-biology, microRNAs orchestrate various cellular functions and have been shown to play critical roles in many processes, including cell differentiation, apoptosis, proliferation and cancer development by acting either as tumour suppressors or oncogenes [25].

The deregulated expression of certain microRNAs has been primarily associated with specific genetic lesions implicated in CLL pathogenesis [26]. However, subsequent evidences collectively suggested that the variability in microRNA expression in CLL can also be due to external stimuli, including those delivered by genotoxic drugs or through the triggering of Toll-like receptor 9 or specific BCRs [27–29]. In particular, the up-regulation of microRNAs from the *miR-132~212* family has been recently associated with BCR triggering, although the functional meaning of this phenomenon has not been yet established [30, 31].

Here, we demonstrated that the engagement of BCR in CLL cells triggers, through the up-regulation of *miR-132*, an inter-chained cascade of events characterized by: i) down-regulation of the deacetylase SIRT1 [32]; ii) increased acetylation of TP53; and iii) up-regulation of TP53 target genes. Consistently, higher *miR-132* constitutive levels were associated with a relative more benign clinical course of patients with M CLL.

## RESULTS

### anti-IgM stimulation up-regulates microRNAs from the *miR-132~212* family

**Figure 1:**
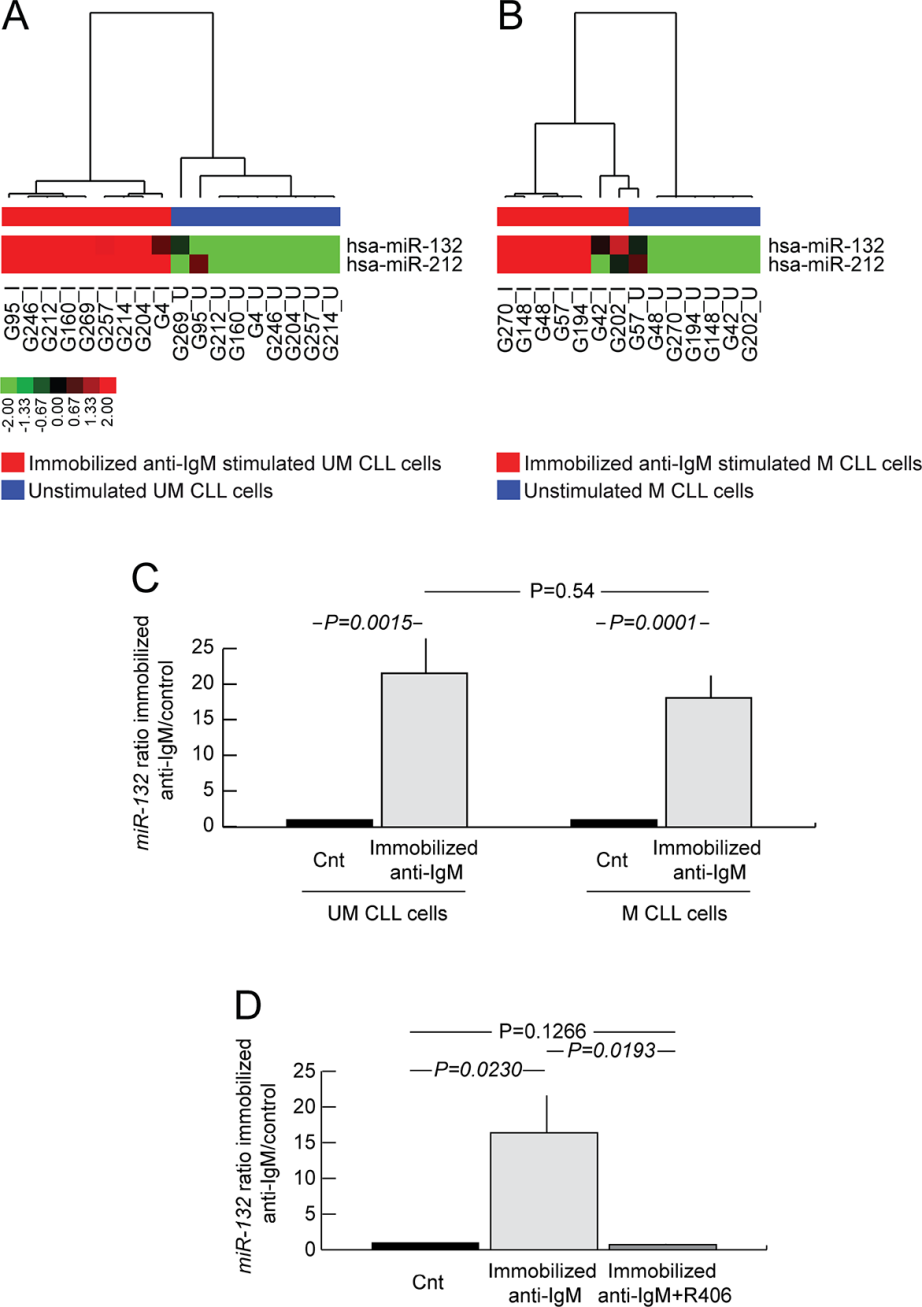
*miR-132* induction upon anti-IgM stimulation of CLL cells **A.** miRome of UM CLL cells upon immobilized anti-IgM stimulation. Hierarchical clustering of immobilized anti-IgM stimulated (red bar under the horizontal dendrogram) and unstimulated (blue bar under the horizontal dendrogram) UM CLL cell samples (9 cases) is shown. Color codes for microRNA expression values refer to mean centered log-ratio values. **B.** miRome of M CLL cells upon immobilized anti-IgM stimulation. Hierarchical clustering of immobilized anti-IgM stimulated (red bar under the horizontal dendrogram) and unstimulated (blue bar under the horizontal dendrogram) M CLL cell samples (7 cases) is shown. Color codes for microRNA expression values refer to mean centered log-ratio values. **C.** qRT-PCR analysis of *miR-132* expression in immobilized anti-IgM stimulated and unstimulated CLL cell samples (12 UM and 16 M). **D.** qRT-PCR analysis of *miR-132* expression in unstimulated CLL cells, or immobilized anti-IgM stimulated, or immobilized anti-IgM plus R406 (4 UM and 4 M). In all graphs data represent mean ± SEM; *P* values refers to Student's *t*-test.

Purified CLL cells from 9 UM CLL and 7 M CLL were either left unstimulated or were stimulated with immobilized or soluble anti-IgM for 20 hours and separately analyzed for changes in their miRome. By applying an identical algorithm and *P* value for supervised analyses, *miR-132* and *miR-212*, belonging to the same microRNA family [33], resulted the sole up-regulated microRNAs both in UM and in M CLL cells upon stimulation with immobilized anti-IgM (Figure 1A–1B). *miR-132* and *miR-212* turned out to be up-regulated upon BCR triggering by immobilized anti-IgM also by analyzing UM and M CLL together (Figure S1), as previously reported [30, 31]. Conversely, no microRNA modulation was observed upon stimulation with soluble anti-IgM (data not shown) in keeping with previous observations comparing the effects of BCR stimulation in CLL by soluble *versus* immobilized anti-IgM [16, 34, 35].

In order to verify the kinetic of *miR-132* induction after anti-IgM stimulation, we performed a time course experiment at various time points in an independent CLL series (13 UM CLL and 17 M CLL). As reported in Figure S2A, *miR-132* expression was transiently induced with a peak at 20 (mean fold change over control 21.7±2.8) hours after stimulation with immobilized anti-IgM. On the contrary, expression of *miR-132* after soluble anti-IgM stimulation showed only a slight up-regulation peaking at 6 hours (mean fold change over control 2.76±1.03; Figure S2A).

Parallel experiments carried out by stimulating purified peripheral blood (PB) normal B cells (*n* = 4) with soluble and immobilized anti-IgM indicated that *miR-132* was up-regulated after 20 hours of BCR stimulation, although with a smaller magnitude compared to immobilized anti-IgM stimulated CLL cells (Figure S2B) [30, 31].

microRNA profile results were validated in CLL cells from a wider series of 28 cases (12 UM and 16 M CLL), in which stimulation with immobilized anti-IgM (hereafter simply indicated as anti-IgM) resulted in a significant induction of *miR-132* expression both in UM (mean fold change over control 21.6±4.9; *P = 0.0015*) and M CLL cells (mean fold change over control 18.2±3.1; *P = 0.0001*), with a comparable magnitude comparing UM and M CLL (*P* = 0.54; Figure 1C).

When CLL cells (4 UM and 4 M CLL) were concomitantly exposed *in vitro* to anti-IgM and the SYK inhibitor R406 [36], the up-regulation of *miR-132* upon anti-IgM stimulation (mean fold change over control 16.3±5.3; *P = 0.023*; Figure 1D) was completely abrogated (mean fold change over control 0.7±0.1; *P* = 0.127; Figure 1D), demonstrating the dependency of *miR-132* up-regulation to BCR triggering.

### anti-IgM stimulation induces changes in mRNA levels of *miR-132* target genes

A parallel GEP, comparing anti-IgM stimulated *versus* unstimulated CLL cells was performed utilizing the same RNA samples used for miRome [14, 28]. In UM CLL, 3,648 differentially expressed genes (1,888 up-regulated and 1,760 down-regulated upon anti-IgM stimulation) were identified (Figure S3A and Table S1), while in M CLL a smaller set of differentially expressed genes (537 genes, 418 up-regulated and 119 down-regulated; Figure S3B and Table S2) was found. Notably, about 75% (387/537 genes) of the latter set of genes was part of the signature connoting the UM CLL category (Figure S3C and Table S1 and Table S2), in keeping with the notion of a more complete response to BCR engagement in UM than in M CLL cells [8, 16, 37]. Again in agreement with these previous studies [8, 37], among the most significant pathways and gene ontology (GO) categories containing differentially expressed genes upon anti-IgM stimulation, both in the UM and M CLL groups, were those related to “Immune Response”, “B-cell receptor signaling pathway” and “Antigen processing and presentation” (Table S3 and Table S4).

With the aim to identify whether microRNAs from *miR-132~212* family were able to affect the gene expression signature of anti-IgM stimulated CLL cells, we retrieved the putative *miR-132* target genes from five different databases (i.e. miRDB, TargetScan, microRNA, GSEA and DIANA-Lab; Table S5) and prior published studied [30]. Given the heterogeneity of the different databases, a merged dataset was identified containing the putative *miR-132* target genes that were present in at least 3 out of 5 databases (167 genes, Table S5). The modulation of these 167 putative *miR-132* target genes in CLL cells upon anti-IgM stimulation was investigated by using a GSEA approach [38].

**Figure 2:**
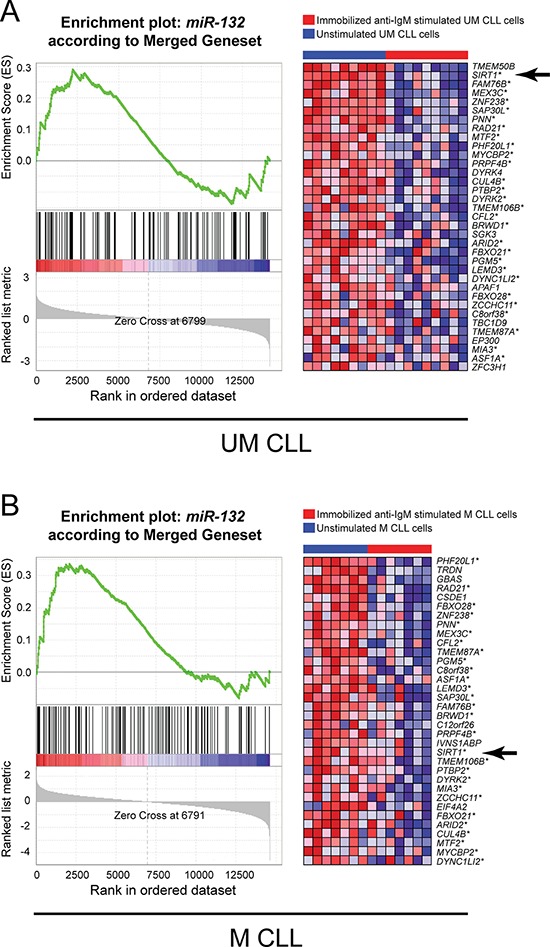
Gene set enrichment analysis (GSEA) of anti-IgM stimulated CLL cells **A.** GEP data of UM CLL cells stimulated with immobilized anti-IgM were tested for an enrichment of the gene set of *miR-132* gene targets, as retrieved from different database for microRNAs targets (see Supplementary Information). GSEA resulted significantly enriched in down-regulated genes in the immobilized anti-IgM stimulated sample group. **B.** GEP data of M CLL cells stimulated with immobilized anti-IgM were tested for an enrichment of the gene set of *miR-132* gene targets, as retrieved from different database for microRNAs targets (see Supplementary Information). GSEA resulted significantly enriched in down-regulated genes in the immobilized anti-IgM stimulated sample group. For each panel, a plot of the Enrichment Score (ES) versus the gene list index is shown on the left; the corresponding heat-map highlighting the relative expression of the gene members belonging to the *miR-132* gene set in immobilized anti-IgM stimulated (red bar) and unstimulated (blue bar) CLL is reported on the right. Asterisks indicate genes in common between UM and M CLL; arrows indicate *SIRT1* gene.

As shown in Figure 2A–2B, anti-IgM stimulation significantly down-regulated 35 and 34 of the 167 putative *miR-132* targets in UM and M CLL cells, respectively, with 28 of the down-regulated *miR-132* target genes being in common to UM and M CLL (identified by asterisks in Figure 2AB, right panels). Since in this context, genes are ranked from top to bottom according to a GSEA correlation score [38], we selected SIRT1 which resulted the top ranked gene among the 28 commonly down-regulated genes in UM CLL. SIRT1 encodes for a deacetalyse, which is a well-known regulator of the p53 pathway in several cell systems [32, 39, 40].

### SIRT1 is a direct target of *miR-132*

**Figure 3:**
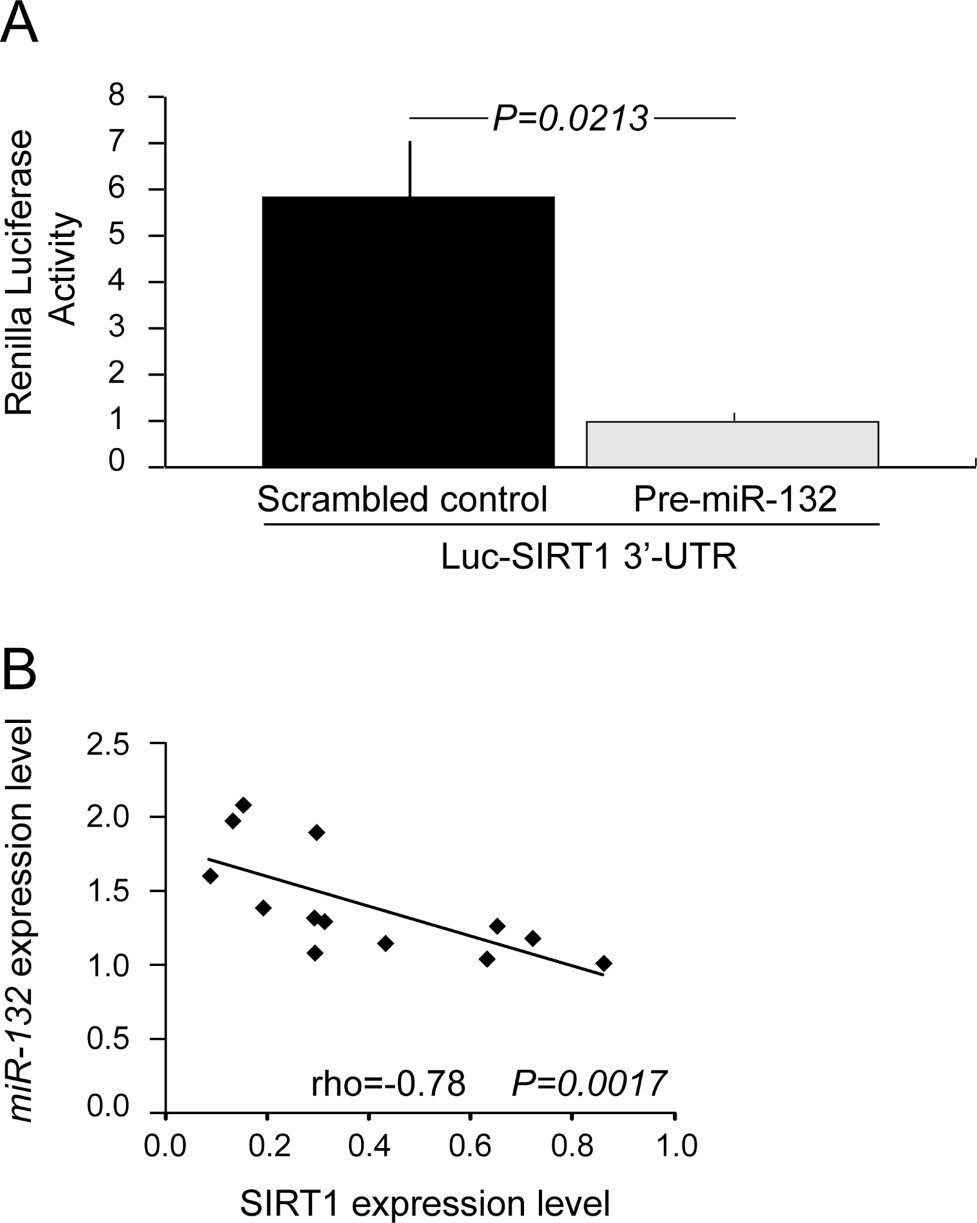
*miR-132* mediates SIRT1 down-regulation **A.** *miR-132* mediates translational repression of *SIRT1* through a binding site in *SIRT1* 3′-UTR. MEC1 cells were cotransfected with pMIRTarget vector containing the 3′-UTR of *SIRT1* (Luc-SIRT1 3′-UTR) and pre-miR-132 (closed histogram) or scrambled control (shaded histogram). Luciferase assay were performed using dual-luciferase assay system after 20-22 hours after transfection. Data represent mean ± SEM of three biological replicates; *P* values refers to Student's *t*-test. **B.** Correlation between *miR-132* and SIRT1 expression in 13 primary CLL samples. Constitutive *miR-132* levels were measured by qRT-PCR. Constitutive SIRT1 protein levels were determined by western blotting. The relative expression of *miR-132* was plotted against that of SIRT1. rho indicates the Spearman's coefficient and *P* value indicates the significance level of correlation.

In-silico analysis identified potential *miR-132* responsive elements in the 3′-UTR of the *SIRT1* gene (Figure S4). When a luciferase reporter construct containing the *SIRT1* 3′-UTR was co-transfected with the pre-miR-132 in cells from the CLL-like cell line MEC1, a significant decrease in luciferase activity (mean fold change over control 0.17±0.03, *P = 0.0213*) was observed with respect to MEC1 cells co-transfected with the *SIRT1* 3′-UTR and the corresponding scrambled control (Figure 3A).

In keeping with these data, an inverse correlation between the constitutive expression levels of *miR-132* and SIRT1 protein levels (rho = −0.78; *P = 0.0017*) was found in primary CLL cells from 7 UM CLL and 6 M CLL patients (Figure 3B).

Collectively, the data obtained from the GEP analysis, *in vitro* transfection assay and ex-vivo correlative studies demonstrate a direct link between *miR-132* and *SIRT1* expression in CLL.

### anti-IgM stimulation associates with down-regulation of SIRT1 protein, increase of TP53 acetylation, and activation of the TP53 pathway

**Figure 4:**
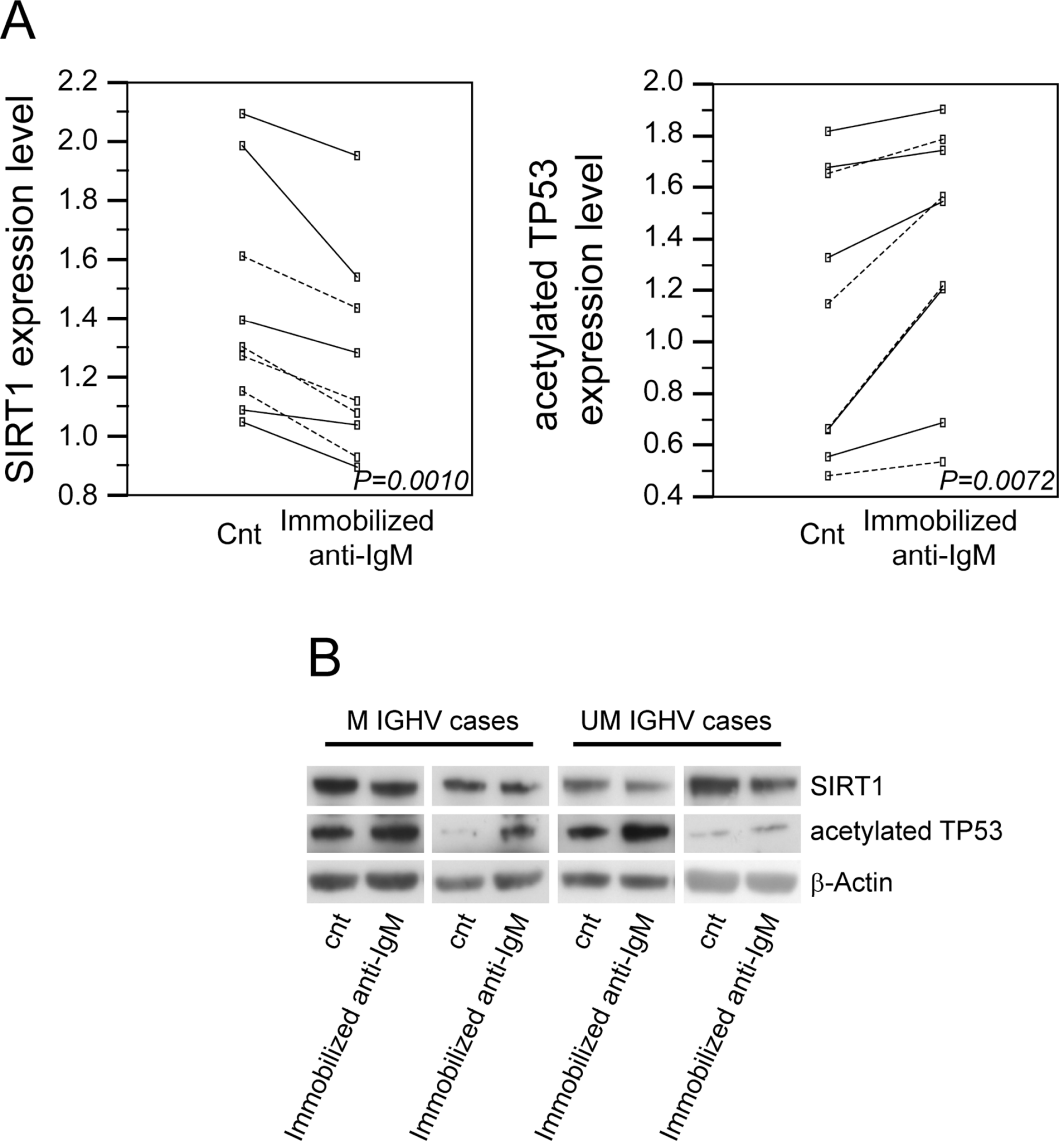
Effects of anti-IgM stimulation of CLL cells on SIRT1 protein levels and TP53 acetylation levels **A.** Dot and line diagrams indicating the changes in SIRT1 protein expression levels or TP53 acetylation levels upon immobilized anti-IgM stimulation are reported as left or right panel, respectively. Unbroken line refers to UM CLL cases and dotted line refers to M CLL cases; *P* values refer to paired *t*-test. **B.** Comparative evaluation of SIRT1 protein levels and TP53 acetylation levels in 4 (2 UM and 2 M) representative CLL cases, as assessed by western blotting. β-Actin levels were used as loading control in all cases.

As shown in Figure 4A–4B, SIRT1 protein levels were significantly reduced (mean fold change 0.87±0.06; *P = 0.001*) upon anti-IgM stimulation of CLL cells (5 UM CLL and 4 M CLL), with similar decrements when comparing UM and M CLL samples (*P* = 0.41). Given the activity of *SIRT1*, a TP53 deacetylase [32, 39, 40], we evaluated the levels of acetylated TP53 upon anti-IgM stimulation in the same CLL samples. In this context, a significant increase in TP53 acetylation was observed (mean fold change 1.21±0.19; *P = 0.0072*; Figure 4A–4B) upon anti-IgM stimulation, again with similar trends between UM and M CLL cell samples (*P* = 0.61).

TP53 acetylation is known to be necessary for activation of the TP53 pathway and up-regulation of TP53 target genes [39, 40]. In keeping with this notion, GEP analysis identified the TP53 target gene *CDKN1A* among the top-ranked genes up-regulated in anti-IgM stimulated CLL cells both in UM and M CLL (Table S1 and Table S2). Moreover, the “TP53 signaling” and “apoptosis” pathway were identified by both the “Pathway-Express” and GO analyses as gene categories that are differentially expressed between unstimulated and anti-IgM stimulated UM and M CLL cells (Table S3 and Table S4).

### *miR-132* enhances apoptosis in EHEB cell line

**Figure 5:**
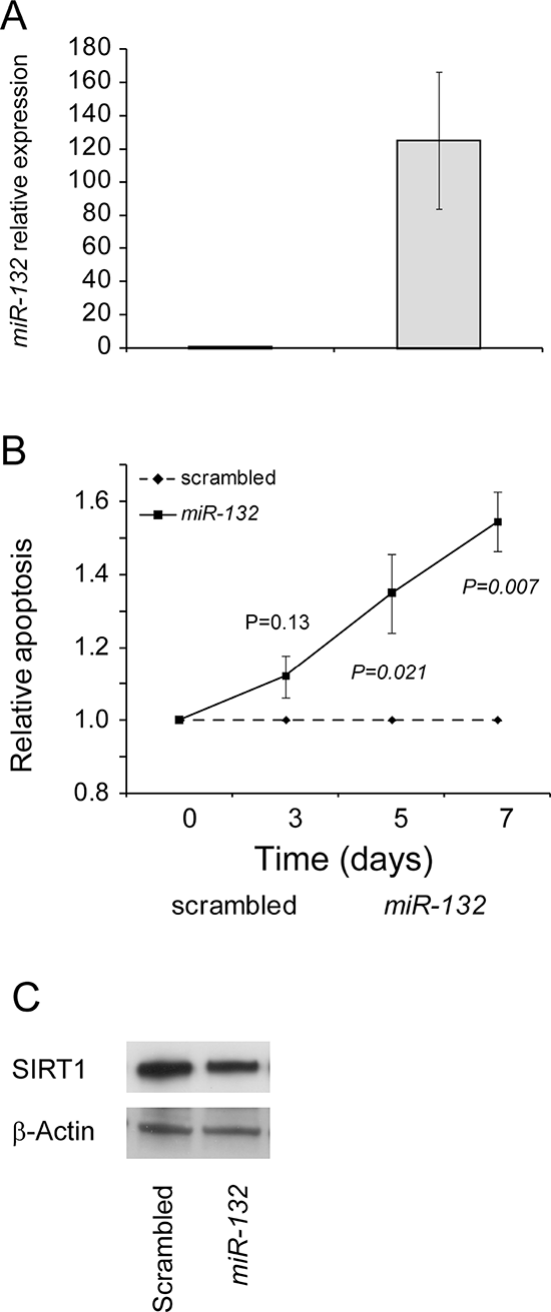
*miR-132* enhances apoptosis and survival in CLL-like EHEB cell line **A.** Effective transfection of *miR-132*. Over-expression of *miR-132* in EHEB cell line transfected with pri-miR-132 compared to the same cell line transfected with scrambled control. **B.** Apoptosis of EHEB cells transfected with *miR-132* or scrambled control. The percentage of apoptotic EHEB cells transfected wit *miR-132* was measured with AnnexinV/7-AAD staining in flow cytometry and normalized to the percentage of EHEB cells transfected with scrambled control. Dotted line indicates scrambled control transfected cells and solid line indicates *miR-132* transfected cells. *P* value (Student's *t*-test) for each time-point are shown. Data represent mean ± SEM of four biological replicates. **C.** *miR-132* over-expression and SIRT1 protein levels. Effects of *miR-132* over-expression on SIRT1 protein levels in EHEB cells. Comparative evaluation of SIRT1 protein levels in EHEB cells, as assessed by western blotting. β-Actin levels were used as loading control in all cases.

The CLL-like EHEB cell line was transfected either with pri-miR-132 or scrambled control and evaluated for apoptosis and viability after 3-5-7 days of culture. qRT-PCR analysis of *miR-132* levels showed that transfection was successful, with an increase in *miR-132* expression levels ranging from 50- to 186-fold compared to cells transfected with scrambled control (Figure 5A). Compared to cells transfected with scrambled control *miR-132*-transfected EHEB cells always showed a greater apoptotic rate which started at day 3 and reached the statistical significance at day 5 (*P = 0.048*) and day 7 (*P = 0.007*) of culture (Figure 5B). Apoptosis was TP53 dependent as witnessed by an early down-regulation of SIRT1 (20 hours) and increased of *CDKN1A* in RNA occurring upon *miR-132* transfection (Figure 5C and data not shown).

### High constitutive expression of *miR-132* identifies an M CLL subset with good prognosis

Expression level of *miR-132* was analyzed by qRT-PCR in an independent series of 134 cases (51 UM CLL and 83 M CLL), all with information available regarding TTT and the main clinical and biological prognostic parameters (Rai stage, *IGHV* mutations, karyotype abnormalities as detected by FISH, CD38, CD49d and ZAP-70 expression; Table S6).

Results of qRT-PCR evaluations showed that constitutive *miR-132* expression levels were variable, ranging from negligible values to values comparable to those found in CLL cells stimulated for 20 hours with anti-IgM (mean = 1.66, range 0.08-11.00 arbitrary unit, a.u.; Figure S5A). Of note, the constitutive levels of *miR-132* in normal PB B cells from 14 healthy donors had similar mean values (1.26 a.u.) but with a narrower variability (range 0.10-2.66 a.u.; Figure S5A) in keeping with results of *in vitro* stimulation experiments (Figure S2B). The variability in constitutive *miR-132* expression values, as detected in CLL cells, was not associated with any of the main biological prognostic markers, including *IGHV* mutation status, high-risk cytogenetic abnormalities or ZAP-70, CD38 and CD49d expression (Figure S5B). On the other hand, all these variables were proven to be significant predictors for TTT in this series (Table S7 and Figure S6), in keeping with previous studies [2, 41].

**Figure 6:**
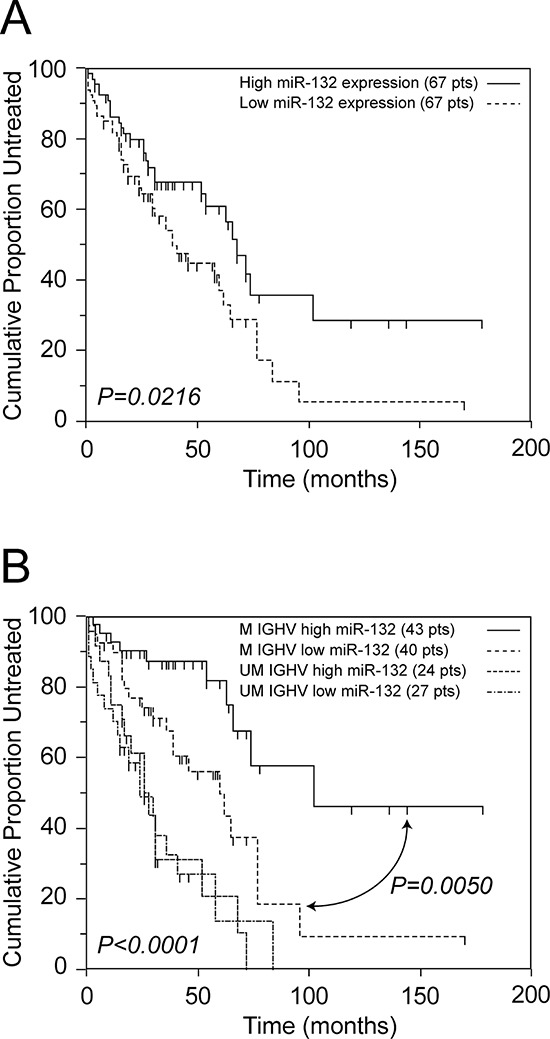
*miR-132* expression levels are associated with a better clinical outcome **A.** Kaplan-Meier curves obtained by comparing TTT intervals of 67 CLL cases with *miR-132* levels above the median value (median TTT 68 months), and with *miR-132* levels below the median value (median TTT 39 months). **B.** Kaplan-Meier curves obtained by comparing TTT intervals of M CLL with *miR-132* levels above the median value (median TTT 100 months), M CLL with *miR-132* levels below the median value (median TTT 60 months), UM CLL with *miR-132* levels above the median value (median TTT 28 months) and UM CLL with *miR-132* levels below the median value (median TTT 28 months). The median relative expression level of the series of 134 CLL cases was 1.27 a.u.. The number of patients (pts) included in each group is reported in parenthesis; the reported *p* value refers to log-rank test.

By setting a cut-off to the median expression value (1.27 a.u.), the median TTT of the 67 CLL patients with *miR-132* expression levels above the cut-off was significantly longer than the median TTT of the 67 cases with *miR-132* expression levels below the cut-off (68 *versus* 39 months, respectively, *P = 0.0216*; Figure 6A and Table S7). However, when the prognostic impact of *miR-132* expression was separately tested in the UM and M CLL subgroups, *miR-132* expression retained its favourable clinical significance only in the context of M CLL. As shown in Figure 6, the median TTT of the 43 M CLL patients with high *miR-132* levels was significantly longer than the median TTT of the 40 M CLL patients with low *miR-132* levels (102 *versus* 60 months, respectively, *P = 0.0050*; Figure 6B), while in the context of UM CLL the median TTT was comparable irrespective of the level of *miR-*132 (*P* = 0.97; Figure 6B). Consistently, multivariate Cox proportional hazard analysis showed that *miR-132* behaved as a favorable marker for TTT prediction both in the whole CLL series (HR = 0.57; *P = 0.0286*) and in the context of M CLL (HR = 0.41; *P = 0.0246*; Table S8 and Table S9), but not in UM CLL (not shown).

## DISCUSSION

In this study, we have examined in detail the miRome changes following the activation of the BCR signaling pathway using anti-IgM as a surrogate for antigen engagement [34]. In particular, confirming previous results [30, 31], we demonstrated that CLL cells, irrespective of their *IGHV* mutational status, respond to BCR stimulation with a clear-cut miRome signature basically characterized by the up regulation of *miR132* and *miR212*, two microRNA belonging to the same cluster [33]. The capacity of the BCR pathway to regulate *miR-132* expression was further validated by demonstrating that up-regulation of *miR-132*: i) requires sustained BCR signaling, as it is significantly higher upon exposure of CLL cells to immobilized compared to soluble anti-IgM, in keeping with literature data on BCR functionality in CLL [16, 34, 35]; ii) is specifically induced by BCR engagement since it is completely abrogated by co-exposing CLL cells to the SYK inhibitor R406 [36]; iii) occurs also in normal B cells either stimulated with soluble or immobilized anti-IgM, although the magnitude of the phenomenon is much lower. The higher variability in the constitutive levels of *miR-132* in circulating CLL cells compared to normal B cell is also consistent with the modulation of *miR-132* expression *in vivo* allegedly due to the continuing antigenic stimulation occurring in CLL tissue sites [6–8].

Several target genes have been described for *miR-132*, including mediators of neurological development, heart pathology, synaptic transmission, inflammation and angiogenesis [42–44]. As shown here in the setting of CLL, and in keeping with other reports [30, 31] *miR-132* turned out to be an important regulator of the gene expression profile induced by anti-IgM stimulation. Of note, among the top-ranked *miR-132* target genes identified in our analysis as down-regulated by anti-IgM stimulation both in UM and M CLL cells, was *SIRT1*, a gene encoding for a class III nuclear deacetylase targeting several histonic and non-histonic proteins including TP53, Ku70, and FOXO [45–48]. A direct functional link between *miR-132* and SIRT1 in CLL was demonstrated by taking advantage of the CLL-like models MEC1 and EHEB, in which an ad-hoc luciferase assay and the transfection of microRNA precursor of *miR-132* clearly showed the capability of *miR-132* to directly down-regulate SIRT1 expression, as also found in other cell systems [45]. The inverse correlation between *miR-132* expression and SIRT1 protein levels that we observed in primary CLL samples, again underlines the close relationship between SIRT1 and *miR-132* in CLL.

In keeping with the evidence that SIRT1 inactivates TP53 by deacetylating a critical lysine residue at position 382 [39], we demonstrated a significant increase of TP53 acetylation at lys382 upon anti-IgM stimulation of CLL cells. This increase, being instrumental in preventing the ubiquitination of lysine residues and the subsequent proteasomal degradation of TP53 [39], represents a pre-requisite for TP53 pathway activation [40]. In agreement with these observations, the TP53 target gene *CDKN1A* resulted significantly up-regulated both in UM and M CLL cells upon anti-IgM stimulation, along with other key genes belonging to the gene ontology categories “apoptosis” and/or “p53 signaling pathway”. Of note, the up-regulation of genes from the TP53 pathway was recently found by Herishanu et al [8] to be associated with the gene expression signature related to BCR pathway activation characteristic of CLL cells from CLL-involved lymph nodes. Moreover, activation of the TP53 pathway upon BCR triggering was also revealed by re-analyzing the GEP data associated with *miR-132* up-regulation reported by Pede et al. [30] (RB, personal communication), and overtly reported by Tavolaro et al. [31], although not associated with the down-regulation of the *miR-132* target *SIRT1*. Finally, using a similar GEP approach, Vallat et al [19] provided evidence for activation of a proapoptotic transcriptional program in CLL cells at later time points after BCR stimulation. In keeping with these data, the ectopic expression of *miR-132* in EHEB cells triggered a TP53-dependent apoptotic pathway, as documented by the early down-regulation of SIRT1 and the up-regulation of *CDKN1A* mRNA. Altogether, these data suggest that activation of apoptotic pathway(s), such as the *miR-132*/SIRT1/TP53 axis, is a common feature of the response of CLL cells to BCR triggering.

Although the role of the activation of the *miR-132*/SIRT1/TP53 pathway in CLL remains obscure, it may represent part of a normal negative feedback mechanism to limit B cell expansion or eliminate autoreactive B cells. In support of this hypothesis are the observations that SIRT1 levels in normal B cells are generally lower than in CLL cells [49], and that BCR stimulation in normal B cells also results in a substantial induction of *miR-132*, as shown here and previously [30, 31]. Moreover, experiments performed with different Eu-TCL1 mouse models resembling progressive/non-anergic and indolent/anergic diseases, as recently published by some of us [50], showed that *miR-132* and *miR-212* expression levels were significantly higher in the neoplastic B cell component of mice with indolent/anergic disease, further supporting the hypothesis of a negative feedback mechanism regulated by miR-132 operating also in this CLL model (S.G. and D.G.E., personal observation).

A number of studies have indicated that signaling through the BCR may be largely responsible for the different clinical course and outcome in patients with CLL expressing either UM or M *IGHV* genes [12, 18, 34, 51]. *In vitro* experiments have reported heterogeneous and somewhat conflicting results regarding the response of CLL cells to anti-IgM stimulation, ranging from increased cell death to suppression of apoptosis and cell proliferation, although the latter response is usually very modest [16, 19, 23, 37, 52–54]. Moreover, besides BCR stimulation, other co-stimulatory signals, allegedly present in a suitable micro-environment *in vivo*, could contribute to induce proliferation and/or provide pro-survival signals to CLL cells [28, 55–62].

**Figure 7:**
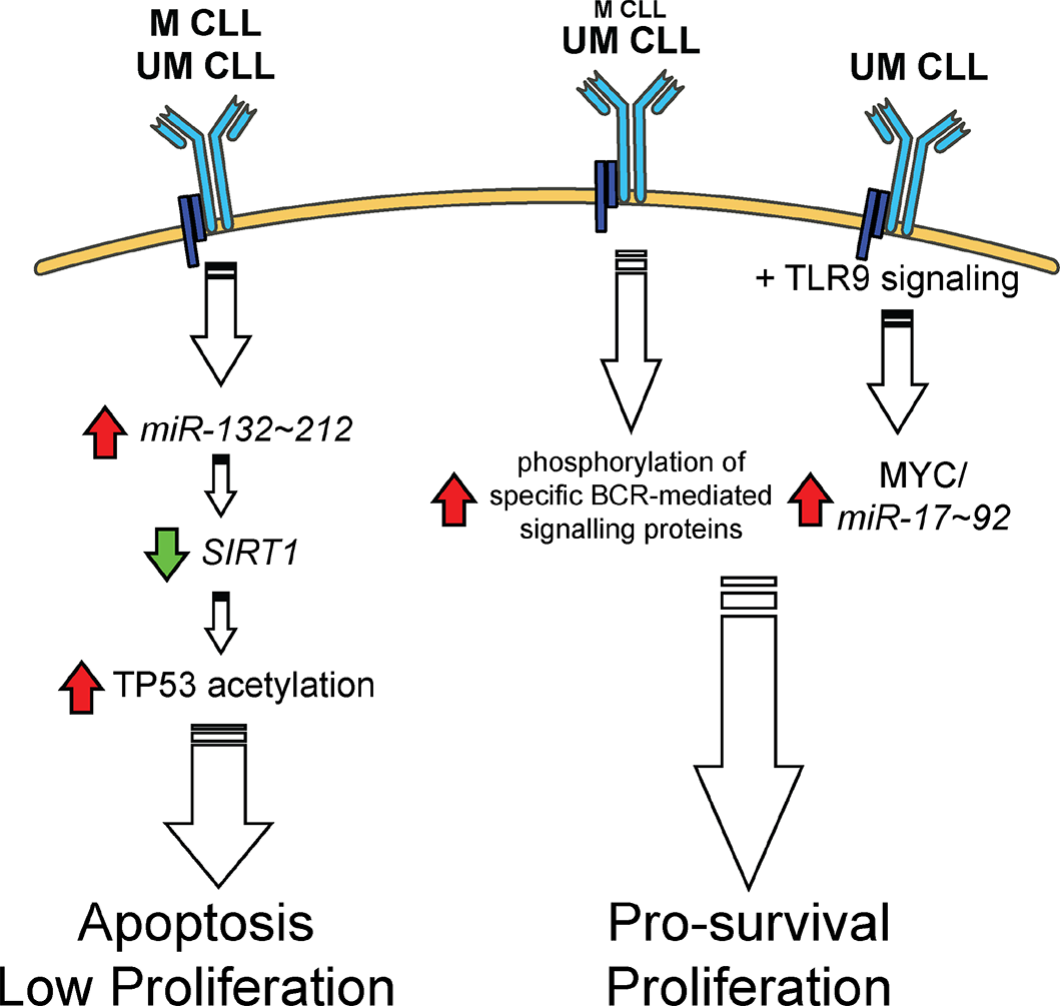
The *miR-132*/SIRT1/TP53 axis and other BCR-related pathways operating in M and UM CLL See text for details. Up-regulations and down-regulations are indicated by a red arrow or a green arrow, respectively.

In the present study, we describe for the first time a complete and inter-chained cascade of events, occurring in both M and UM CLL cells, triggered by BCR engagement via *miR-132* up-regulation and sequentially characterized by SIRT1 down-regulation, TP53 acetylation and TP53 pathway activation. It can be speculated that this *miR-132*/SIRT1/TP53 axis represents the prevalent pathway activated in M CLL cells upon BCR triggering *in vivo*, while in UM CLL cells the activity of this pathway is overcome by additional pathways triggered by other types of antigens or other exogenous stimuli that may transmit more efficient pro-survival and/or proliferative signals to these cells (Figure 7) [28, 55–62]. The availability of such signals, or the capacity of the CLL cells, expressing either a UM or M IGHV gene, to differently respond to these external stimuli could provide a putative explanation for the different biological and clinical behavior of the UM and M CLL subsets (Figure 7). The finding that high levels of *miR-132* are associated with a less aggressive clinical behaviour in the context of M CLL but not in UM CLL is in keeping with this reasoning.

Currently, several inhibitors of kinases downstream the BCR have been developed in clinical trials, and have emerged as promising novel therapies for CLL patients. These agents include inhibitors of SYK, BTK and PI3Kδ [11, 63–65]. Typically, these agents operate by thwarting the BCR signal as well as signals activated in response to other ligands that mediate the cross-talk between CLL cells and the tumour microenvironment [63, 65]. Recently, differences emerged regarding the capability of CLL to respond to BTK inhibitors [66], and, in particular, M CLL patients may have an inferior response when compared to patients with UM CLL [64]. In this context, the up-regulation of *miR-132* upon BCR triggering and its specific abrogation upon exposure to the SYK inhibitor R406, as reported here, opens the possibility that in some instances, e.g. in M CLL, the down-regulation of the *miR-132*/SIRT1/TP53 axis by BCR inhibitors may paradoxically attenuate the clinical effects of BCR inhibitors in CLL.

## METHODS

### Primary cells from CLL patients and healthy donors

The study included PB samples from 178 CLL patients, divided as follows: i) a discovery panel of 16 CLL cases utilized for global microRNA expression profile (miRome) and gene expression profiling (GEP) analyses; ii) a validation panel of additional 28 CLL cases utilized for functional assays; iii) a third panel of 134 CLL cases, with Time-To-first-Treatment (TTT) intervals available [67], utilized for clinical correlation studies. All patients provided informed consent in accordance with local Institutional Review Board requirements (IRB-04-2010, Centro di Riferimento Oncologico, Aviano, Italy) and declaration of Helsinki. PB mononuclear cells were separated by Ficoll gradient centrifugation (Amersham Biosciences, Uppsala, Sweden). Detection of *IGHV* mutational status was performed as previously reported [29]. The 2% cutoff was chosen to discriminate UM *versus* M CLL cases. *IGHV* mutational status and additional biological features of CLL cases entering this study, including expression of CD38, CD49d, ZAP-70, interphase fluorescence in-situ hybridization (FISH) for the main chromosomal abnormalities, Rai stage, and the experiments for which each sample has been used are listed in Table S6 [1, 29, 41].

The purity of the CLL cells, as monitored by flow-cytometry, always exceeded 95% of clonal CD5^+^/CD19^+^ cells. Whenever necessary, CLL cells were purified by negative selection using anti-CD3, anti-CD14 and anti-CD16 mouse monoclonal antibodies and Dynabeads coated with a pan anti-mouse IgG antibody (Dynal Biotech, Oslo, Norway) [56].

Normal B cells from healthy donors (*n* = 18) were obtained from peripheral blood samples by Ficoll- gradient centrifugation and used either directly or cryopreserved until use. All studies were performed on highly purified cells (>95% pure), as results of negative selection by immunomagnetic beads when required [68].

### Cell culture conditions

Highly purified CLL cells and normal B cells were cultured (1 × 10^7^ cells/ml) in RPMI-1640 supplemented with 10% heat-inactivated fetal bovine serum, 100 U/ml penicillin, 0.1 mg/ml streptomycin, 2 mM L-glutamine and 1 mM sodium pyruvate (Life Technologies, Carlsbad, CA, USA). CLL cells were stimulated, according to previous studies [16, 36], at various time points with 10 μg/ml goat F(ab')2 anti-human IgM (soluble anti-IgM) (SouthernBiotech, Birmingham, AL) or with 1 × 10^7^/mL Dynabeads M-450 Epoxy (Life Technologies) coated with 10 μg goat anti-human IgM (immobilized anti-IgM) (SouthernBiotech, Birmingham, AL) [56]. The coating procedure was done according to the manufacturer's instructions (Life Technologies). In selected experiments, CLL cells were co-stimulated with immobilized anti-IgM and cultured in the presence of R406 4 μM (Axon Medchem, The Netherlands) as previously described [36].

### miRome, GEP and data mining tools

Total RNA was extracted from purified CLL cells using the TRIZOL Reagent (Life Technologies) and validated for integrity and purity using the Agilent 2100 Bioanalyzer (Agilent Technologies, Santa Clara, CA).

Single-color hybridization microarray experiments for miRome were performed with 100 ng total RNA/sample labeled with Cyanine(Cy)-3 dye using the microRNA Complete Labeling System & Hyb Kit (Agilent Technologies). Cy3-labeled RNA was hybridized to the Human microRNA microarray Version 3 from the Sanger database v12.0 (Agilent Technologies).

GEP was performed using the Whole Human Genome (4×44K) oligo microarray platform (Agilent Technologies) as previously described [28, 29]. Microarray slides were analyzed with an Agilent Microarray Scanner (Agilent Technologies).

The hybridization signal values for the multiple probes were obtained with the use of Agilent Feature Extraction Software 10.7.3 (Agilent Technologies). Microarray data are available in Gene Expression Omnibus (GEO; http://www.ncbi.nlm.nih.gov/geo/) under accession number GSE52776.

Bioinformatics analyses were performed using GeneSpringGX 11.5 (Agilent Technologies). Results were visualized by hierarchical clustering applying Ward's method with Euclidean distance.

The specific targets for *miR-132* targets were identified by taking advantage of the following five datasets for putative microRNA targets: i) GSEA (http://www.broadinstitute.org/gsea/msigdb/cards/GACTGTT,MIR-212,MIR-132.html); ii) microRNA (http://www.microrna.org/microrna/home.do); iii) miRDB (http://mirdb.org/miRDB/index.html); iv) Targetscan (http://www.targetscan.org/vert_50/); v) DIANA (http://diana.cslab.ece.ntua.gr/microT/). In particular, among 959 putative *miR-132* target genes overall comprised in the above quoted five datasets (Table S5), we selected those shared by at least three out of five datasets; this resulted in the identification of 167 putative *miR-132* target genes that were up-loaded to the Gene Set Enrichment Analysis (GSEA) software [38], for subsequent analyses (Table S5). Further details are provided in Supplementary Information.

### Quantitative-real-time polymerase chain reactions (qRT-PCR)

Expression of selected microRNAs and of the control *RNU6B* was assessed using a standard TaqMan MicroRNA assay kit (Life Technologies) according to the manufacturer's instructions and as previously described [28, 29]. Briefly, microRNA was reverse transcribed to cDNA using gene-specific primers and the relative amount of each microRNA was computed using the equation 2^−ΔCt^, where ΔCt = (Ct _microRNA_ – Ct _RNUB6_).

### Luciferase assay

Cells from the CLL-like cell line MEC1 (0.5–1 × 10^5^ cells per well) were seeded in 96-well plates. After 24 hours, 100 ng pMIRTarget vector containing the 3′-UTR of *SIRT1* (OriGene Technologies, Rockville, MD), 10 ng Renilla vector (Promega Corporation, Madison, WI), and 100 ng pre-miR-132 or 100 ng scrambled control oligonucleotides (Life Technologies) were transfected using Fugene HD transfection reagent (Promega Corporation) according to the manufacturer's instructions. Luciferase assays were performed using the dual-luciferase assay system (Promega Corporation) 20-22 hours after transfection.

### Transfection

Three micrograms of microRNA precursor for *miR-132* (Ambion, TX) were transfected into 5 × 10^6^ CLL-like cell line EHEB cells with the Amaxa Nucleofector system (Lonza Cologne GmbH, Germany) according to manufacturer's guidelines. As negative control, cells were transfected with equal amounts of pre-miR-negative control#1 (Ambion).

### Western blot

Total proteins were extracted from EHEB or CLL cells collected 20 hours after transfection or co-stimulation with immobilized anti-IgM, loaded and run in NuPAGE Novex 4-12% Bis-Tris gels (Life Technologies) prior to transfer to nitrocellulose membranes (GE Healtcare, UK) for Western Blot analysis and detection by ECL (GE Healtcare) or Immobilon (Millipore Corporation, MA). Rabbit-anti-SIRT1 (C14H4) (Cell Signaling Technology, Danvers, MA), Rabbit-anti-acetyl-p53 (Lys382) (Cell Signaling Technology) antibodies (final dilution 1:1000) were used for protein detection. Anti-beta Actin antibody (AC-15) (HRP) (Abcam, UK) was used for loading control (final dilution 1:100.000). Densitometric quantitation of western blots was determined with the Quantity One 4.1.0 software (Bio-Rad).

### Functional studies

MicroRNA-transfected EHEB cells were cultured in RPMI-1640 for 7 days. The percentage of apoptotic cells was determined by AnnexinV and 7-amino-actinomycin-D (7-AAD) (Becton-Dickinson, San Jose, CA) staining. Data were acquired on a FACS Canto flow cytometer and analysed by the FACS Diva software (Becton-Dickinson).

### Statistical analysis

Clinical correlations, performed with the MedCalc v9.5 software, were made using Kaplan-Meier plots and log-rank test; TTT was chosen as clinical endpoint. The Cox proportional hazard regression model was used to assess the independent effect of co-variables, treated as dichotomous on TTT, with a stepwise procedure for selecting significant variables.

## SUPPLEMENTAL METHODS




















