# Supplementary material for: The SIRT1/TP53 axis is activated upon B-cell receptor triggering via miR-132 up-regulation in chronic lymphocytic leukemia cells
Source: Oncotarget. 2015 May 11;6(22):19102–17. doi: 10.18632/oncotarget.3905 (PMC4662478; doi:10.18632/oncotarget.3905)
Supplement: Supplementary file 1 [file oncotarget-06-19102-s001.pdf]

SUPPLEMENTAL METHODS

MicroRNA Expression Profiling (miRome) data mining tools

MicroRNA data were analyzed using the GeneSpring GX 11.5 software (Agilent Technologies, Santa Clara, CA); pre-processing and pre-filtering steps were carried out according to Agilent instruction. To identify the differentially expressed microRNAs, *t* statistic was applied and *P* values were then adjusted for multiple testing with Benjamini and Hochberg's method to control the false discovery rate. Differentially expressed microRNAs were identified according to an adjusted *P* value ≤ 0.001. Expression profile results were visualized using the Cluster and Tree View programs (Eisen Laboratory, Stanford University) [1].

Gene Expression Profiling (GEP) data mining tools

GEP data were analyzed using the GeneSpring GX 11.5 software (Agilent Technologies, Santa Clara, CA); pre-processing and pre-filtering steps were carried out according to Agilent instruction. To identify the differentially expressed genes, t statistic was applied and *P* values were then adjusted for multiple testing with Benjamini and Hochberg's method to control the false discovery rate. Differentially expressed genes were identified according to an adjusted *P* value ≤ 0.01 and a fold change at least equal to 1.5. GEP results were visualized using the Cluster and Tree View programs (Eisen Laboratory, Stanford University) [1]. The biological functions of genes were investigated using Onto-Tools [2]. Significant Gene Ontology (GO) categories and pathway differentially expressed were selected for having a *P* value of at least 0.05, and containing at least 6 genes per category. Gene Set Enrichment Analysis (GSEA) [3–6] was used to identify the putative microRNAs involved in gene deregulation from the online database available at the GSEA Web site (http://www.broadinstitute.org/gsea/). In addition, selected gene sets, as retrieved from different database for putative microRNA targets (http://www.broadinstitute.org/gsea/msigdb/cards/GACTGTT,MIR-212,MIR-132.html, http://www.microrna.org/microrna/home.do, http://www.targetscan.org/vert_50/, http://diana.cslab.ece.ntua.gr/microT/ and http://mirdb.org/miRDB/index.html), were uploaded to GSEA for inclusion in the analysis. Enriched or over-represented genes sets between immobilized anti-IgM stimulated or unstimulated CLL cells were identified using 1.000 permutations of the phenotype labels.

REFERENCES

1. Eisen MB, Spellman PT, Brown PO, Botstein D. Cluster analysis and display of genome-wide expression patterns. Proc Natl Acad Sci U S A. 1998; 95:14863–8.
2. Draghici S, Khatri P, Martins RP, Ostermeier GC, Krawetz SA. Global functional profiling of gene expression. Genomics. 2003; 81:98–104.
3. Bomben R, Gobessi S, Dal BM, Volinia S, Marconi D, Tissino E, Benedetti D, Zucchetto A, Rossi D, Gaidano G, Del PG, Laurenti L, Efremov DG, et al. The miR-17-92 family regulates the response to Toll-like receptor 9 triggering of CLL cells with unmutated IGHV genes. Leukemia. 2012.
4. Bomben R, Dal-Bo M, Benedetti D, Capello D, Forconi F, Marconi D, Bertoni F, Maffei R, Laurenti L, Rossi D, Del Principe MI, Luciano F, Sozzi E, et al. Expression of mutated IGHV3-23 genes in chronic lymphocytic leukemia identifies a disease subset with peculiar clinical and biological features. Clin Cancer Res. 2010; 16:620–628.
5. Mootha VK, Lindgren CM, Eriksson KF, Subramanian A, Sihag S, Lehar J, Puigserver P, Carlsson E, Ridderstrale M, Laurila E, Houstis N, Daly MJ, Patterson N, et al. PGC-1alpha-responsive genes involved in oxidative phosphorylation are coordinately downregulated in human diabetes. Nat Genet. 2003; 34:267–273.
6. Subramanian A, Tamayo P, Mootha VK, Mukherjee S, Ebert BL, Gillette MA, Paulovich A, Pomeroy SL, Golub TR, Lander ES, Mesirov JP. Gene set enrichment analysis: a knowledge-based approach for interpreting genome-wide expression profiles. Proc Natl Acad Sci U S A. 2005; 102:15545–15550.

SUPPLEMENTARY FIGURES AND TABLES

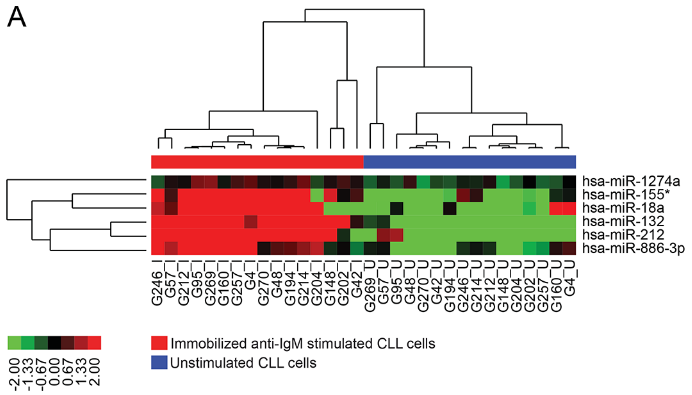

Supplementary Figure S1: *miR-132* induction upon anti-IgM stimulation of CLL cells. miRome of CLL cells upon immobilized anti-IgM stimulation. Hierarchical clustering of immobilized anti-IgM stimulated (red bar under the horizontal dendrogram) and unstimulated (blue bar under the horizontal dendrogram) CLL cell samples (16 cases) is shown. Color codes for microRNA expression values refer to mean centered log-ratio values.

A

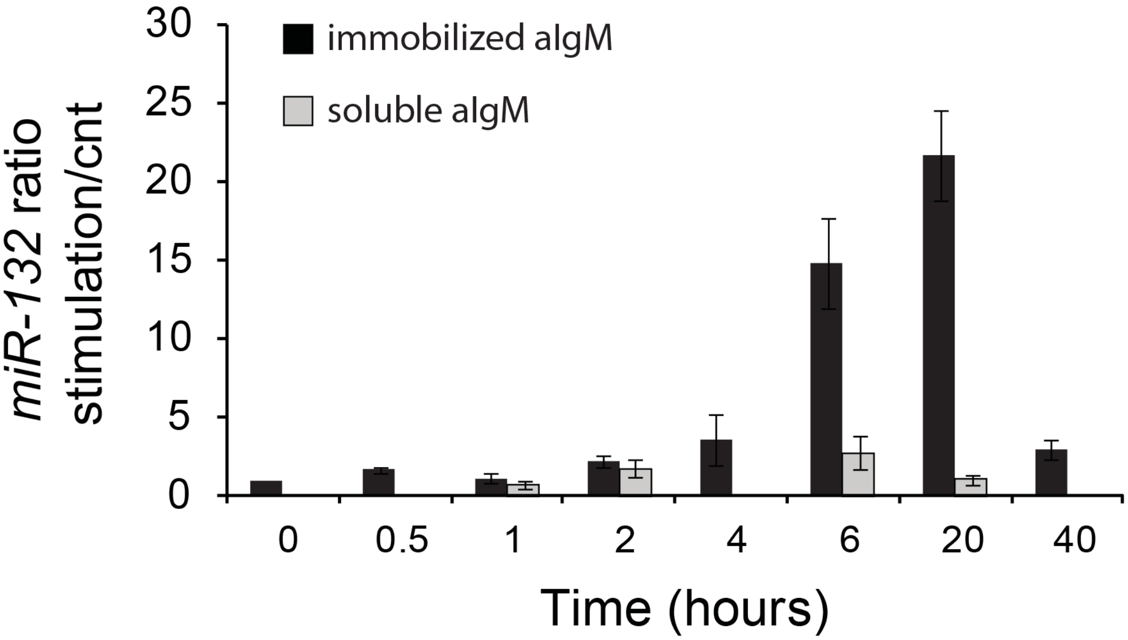

B

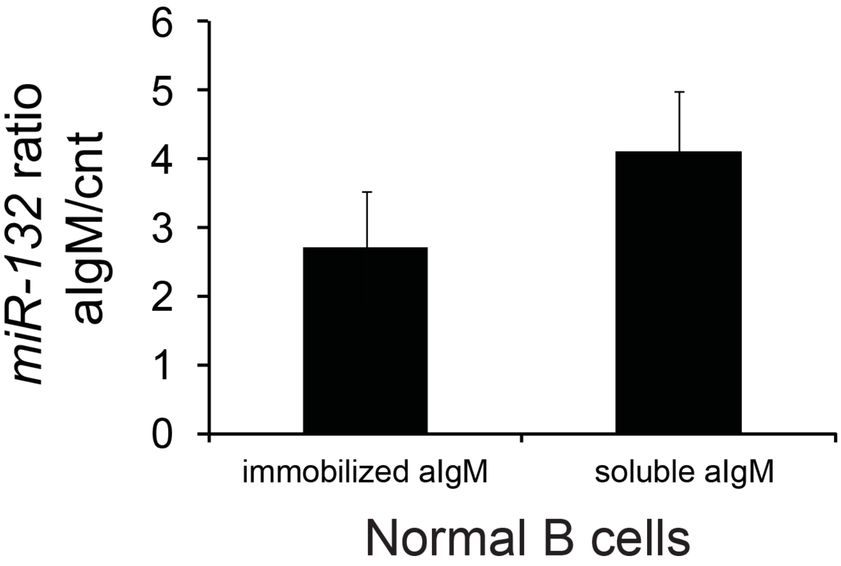

Supplementary Figure S2: Time course expression of *miR-132* upon anti-IgM stimulation. A. qRT-PCR analysis of *miR-132* expression levels following soluble and immobilized anti-IgM stimulation of CLL cells (13 UM CLL and 17 M CLL). **B.** qRT-PCR analysis of *miR-132* expression levels following soluble and immobilized anti-IgM stimulation for 20 hours of normal B cells from healthy donors (n = 4). In all graphs data represent mean ± SEM.

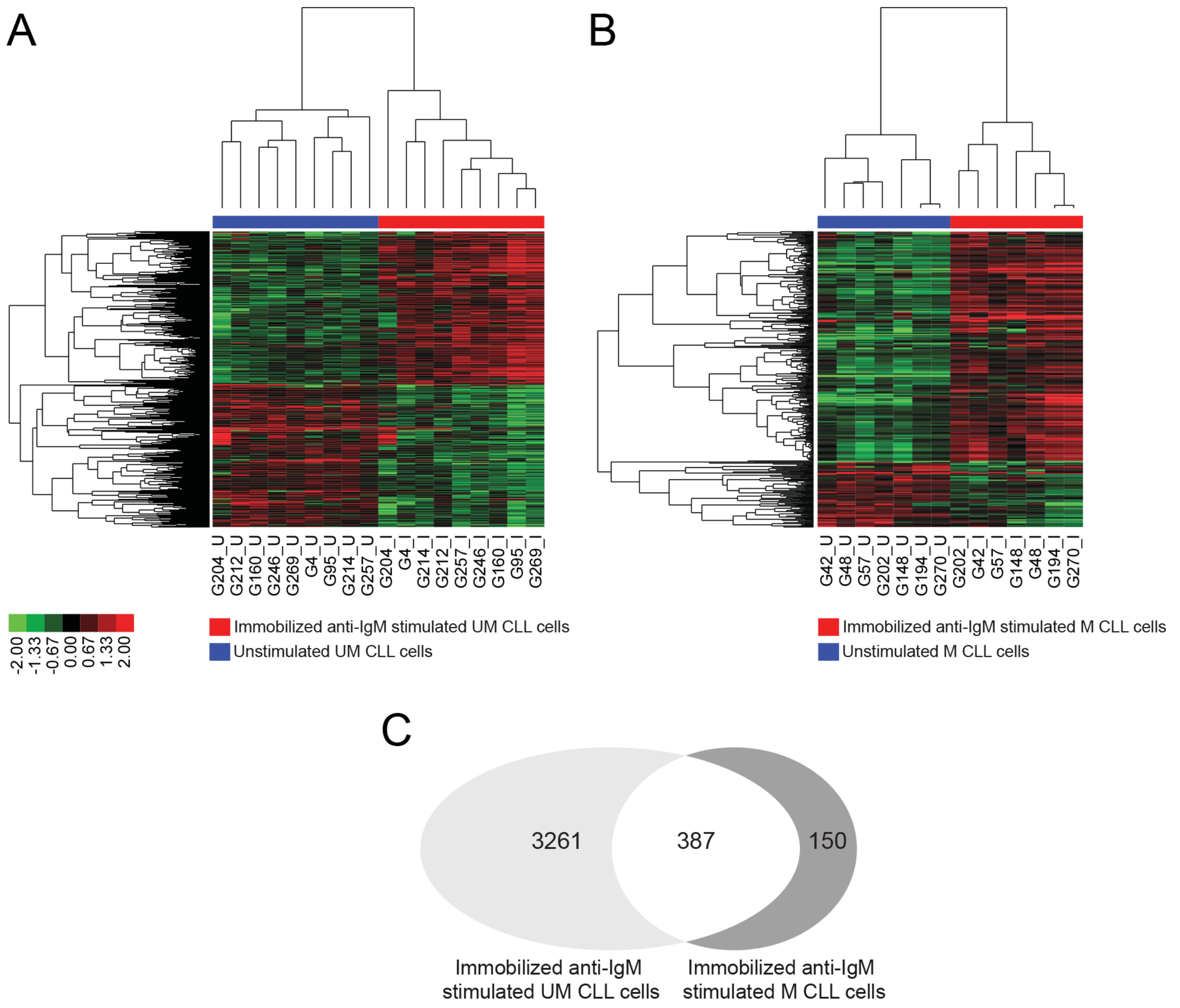

Supplementary Figure S3: GEP upon anti-IgM stimulation of CLL cells. A. GEP of UM CLL cells upon immobilized anti-IgM stimulation. Hierarchical clustering of immobilized anti-IgM stimulated (red bar under the horizontal dendrogram) and unstimulated (blue bar under the horizontal dendrogram) UM CLL cell samples (9 cases), using the 3, 648 differentially expressed genes, is shown. Color codes for gene expression values refer to mean centered log-ratio values. **B.** GEP of M CLL cells upon immobilized anti-IgM stimulation. Hierarchical clustering of immobilized anti-IgM stimulated (red bar under the horizontal dendrogram) and unstimulated (blue bar under the horizontal dendrogram) M CLL cell samples (7 cases), using the 537 differentially expressed genes, is shown. Color codes for gene expression values refer to mean centered log-ratio values. **C.** Venn diagram showing the number of genes in common between the genes differentially expressed in anti-IgM stimulated *versus* unstimulated UM CLL (3.648 genes), cells and the genes differentially expressed in anti-IgM stimulated *versus* unstimulated M CLL cells (537 genes).

```
     3'gcugguaccgACAUCUGACAAu 5' miR-132
                 ||  ||||||||
1600:5'auuuuuacagUGAAGACUGUUu 3' SIRT1

     3'gcugGUAC-CGACAUCUGACAAu 5' miR-132
              :|||   :|||||||||||
1665:5'ggcaUAUGUUUUGUAGACUGUUu 3' SIRT1

     3'gcugguaccGACAUCUGACAAu 5' miR-132
                :||||  ||||||
1732:5'guuuuuuacUUGUACACUGUUu 3' SIRT1
```

http://www.microrna.org/microrna/getGeneForm.do

Supplementary Figure S4: *miR-132*/SIRT1 alignment. The alignment between *miR-132* and *SIRT1* is retrieved from http://www.microrna.org/microrna/home.do.

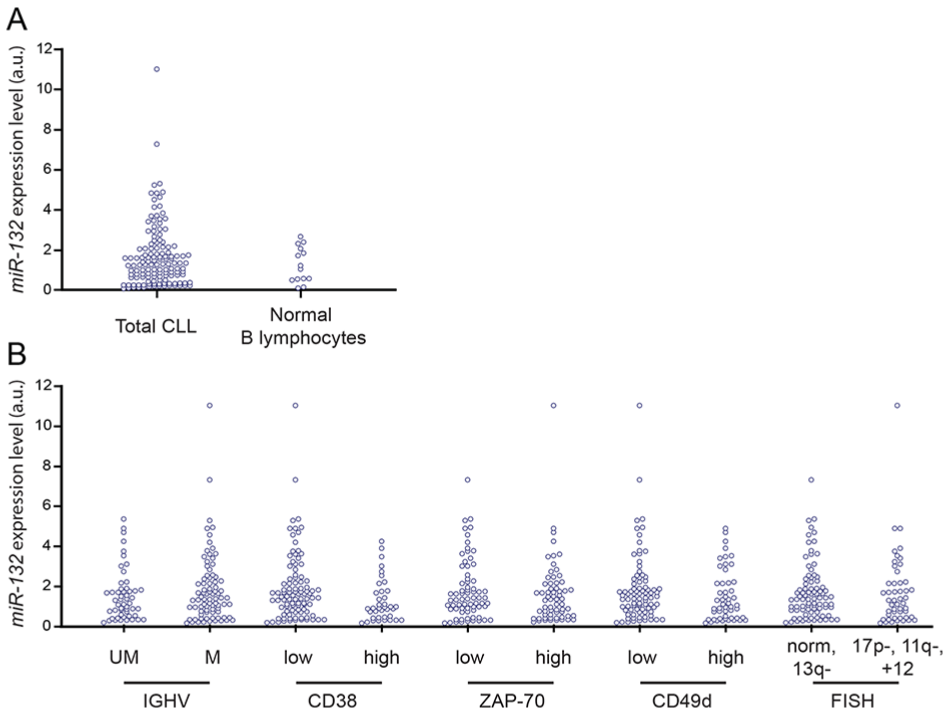

Supplementary Figure S5: Constitutive expression of *miR-132* in CLL. A. Dot plots showing the constitutive expression of *miR-132* in CLL cases and normal B cells from healthy donors. **B.** Dot plots showing the constitutive expression of *miR-132* in CLL cases split according to *IGHV* mutational status (UM, unmutated IGHV status *versus* M, mutated IGHV status), CD38, ZAP-70, CD49d expression (low and high refer to above and below the established cut-off, respectively), and chromosomal alterations as investigated by interphase FISH (normal karyotype, norm, and 13q- *versus* 17p- or 11q- or 12+). a.u. means arbitrary units.

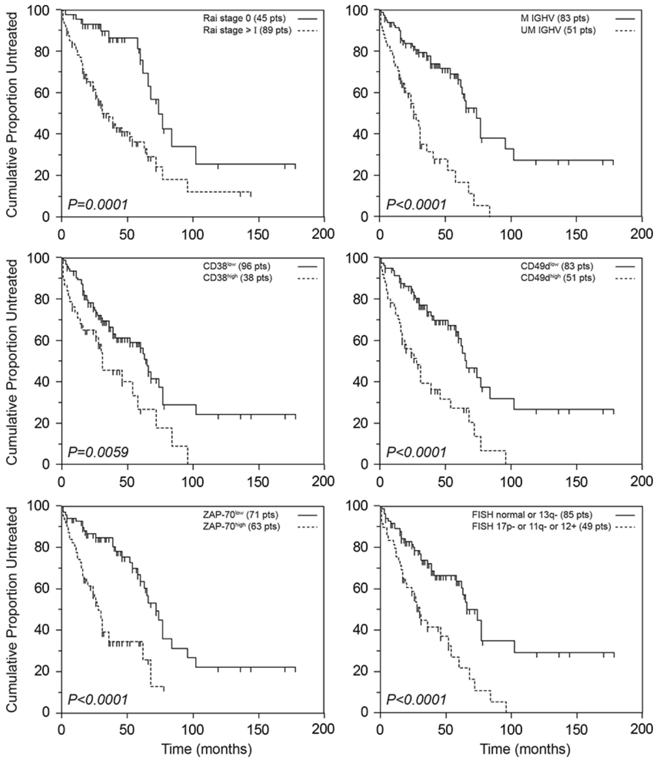

Supplementary Figure S6: Prognostic impact of clinical and biological prognosticators on CLL patients. Kaplan-Meier curves obtained by comparing TTT of CLL patients split according to Rai stage at diagnosis (Rai 0 *versus* I-V), *IGHV* mutational status (UM, unmutated IGHV status *versus* M, mutated IGHV status), CD38, ZAP-70, CD49d expression (expression above, high in apex, *versus* expression below, low in apex, the established cut-offs), and chromosomal alterations as investigated by interphase FISH (17p- or 11q- or 12+ *versus* normal karyotype and 13q-). The numbers of patients (pts) included in each group are reported in parenthesis; the reported *P* values refer to log-rank test.

Supplementary Table S1: Differentially expressed genes between immobilized anti-IgM stimulated and unstimulated UM CLL cells.

Supplementary Table S2: Differentially expressed genes between immobilized anti-IgM stimulated and unstimulated M CLL cells.

Supplementary Table S3: Differentially represented pathways in CLL cells stimulated with immobilized anti-IgM *versus* CLL cells left unstimulated.

Supplementary Table S4: GO categories differentially represented in CLL cells stimulated with immobilized anti-IgM *versus* CLL cells left unstimulated.

Supplementary Table S5: *miR-132* targets retrived by the different database.

Supplementary Table S6: Biological features of CLL cases.

Supplementary Table S7: Univariate Cox regression analyses of TTT.

Supplementary Table S8: Multivariate Cox regression analyses of TTT.

Supplementary Table S9: Univariate Cox regression analyses of TTT in M */IGHV* CLL cases.
